# tmChem: a high performance approach for chemical named entity recognition and normalization

**DOI:** 10.1186/1758-2946-7-S1-S3

**Published:** 2015-01-19

**Authors:** Robert Leaman, Chih-Hsuan Wei, Zhiyong Lu

**Affiliations:** 1 National Center for Biotechnology Information, 8600 Rockville Pike, Bethesda, Maryland 20894, USA

## Abstract

Chemical compounds and drugs are an important class of entities in biomedical research with great potential in a wide range of applications, including clinical medicine. Locating chemical named entities in the literature is a useful step in chemical text mining pipelines for identifying the chemical mentions, their properties, and their relationships as discussed in the literature.

We introduce the tmChem system, a chemical named entity recognizer created by combining two independent machine learning models in an ensemble. We use the corpus released as part of the recent CHEMDNER task to develop and evaluate tmChem, achieving a micro-averaged f-measure of 0.8739 on the CEM subtask (mention-level evaluation) and 0.8745 f-measure on the CDI subtask (abstract-level evaluation). We also report a high-recall combination (0.9212 for CEM and 0.9224 for CDI). tmChem achieved the highest f-measure reported in the CHEMDNER task for the CEM subtask, and the high recall variant achieved the highest recall on both the CEM and CDI tasks.

We report that tmChem is a state-of-the-art tool for chemical named entity recognition and that performance for chemical named entity recognition has now tied (or exceeded) the performance previously reported for genes and diseases. Future research should focus on tighter integration between the named entity recognition and normalization steps for improved performance.

The source code and a trained model for both models of tmChem is available at: http://www.ncbi.nlm.nih.gov/CBBresearch/Lu/Demo/tmChem. The results of running tmChem (Model 2) on PubMed are available in PubTator: http://www.ncbi.nlm.nih.gov/CBBresearch/Lu/Demo/PubTator

## Background

The effects of chemicals on living systems of every scale make them an exceptionally important class of entities for biomedical research and clinical applications. These effects may be therapeutic (as in drugs), investigational (as in drug discovery) or unintentional (as in adverse effects or environmental toxicities) [1]. As such, chemicals/drugs are one of the topics most frequently searched in PubMed [2,3]. While extracting chemical mentions from biomedical literature has been attempted previously [4], the task has not yet yielded results approaching those of better-studied entity types such as genes/proteins [5-7], species [8], and diseases [9]. This is likely due in part to both the great variety of biologically relevant chemical structures and to the somewhat different properties exhibited by chemical mentions. These properties include systematic and semi-systematic methods for describing chemical structure (e.g. formulas and IUPAC names), whose highly compositional nature makes it difficult to precisely determine the entity boundaries, or even the number of entities present.

### Related work

Recent reviews detail the considerable previous work on chemical text mining from the biomedical or chemical literature, concentrating primarily on chemical named entity recognition [10,11]. Many of these previous systems focus effort on a specific area of interest. For example, the Jochem dictionary uses a lexical approach to identify drugs and other small molecules named in biomedical text [12], while Klinger, et al. [13] use a machine learning approach with conditional random fields (CRF) to find chemicals mentioned using the IUPAC systematic format, reporting an f-measure of 0.815.

Systems with a broader focus have also been developed. The OSCAR system employs a hybrid approach for chemical text mining in chemistry publications [14]. Rocktaschel, et al. [4] use a lexical approach to locate trivial names, abbreviations and drugs, and also employ a conditional random field method to locate IUPAC names to create a hybrid chemical named entity recognition tool called ChemSpot, reporting an f-measure of 0.681 on the SCAI corpus.

There has also been increasing interest in the creation of annotated corpora to assist in system development and evaluation. Kolarik, et al. [15] survey available chemical resources and also report the creation of the SCAI corpus with chemical mention annotations. In addition, the CALBC Silver Standard corpus - created by pooling the output of multiple systems - includes one mention type which combines chemicals and drugs [16,17].

The most recent effort to create annotated corpora to support chemical named entity recognition was the CHEMDNER task at BioCreative IV, which attracted 27 participating teams [18]. This paper describes the creation of the tmChem system, our submissions to the CHEMDNER task [19], and subsequent experiments and analysis. One of our submissions achieved the highest f-measure reported for the CEM subtask, and our high recall variant achieved the highest recall reported in both the CDI and CEM subtasks.

## Methods

### CHEMDNER dataset

**Figure 1:**
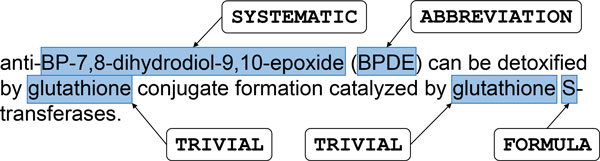
**Sample sentence from the CHEMDNER Corpus with illustrative chemical entity annotations of types SYSTEMATIC, ABBREVIATION, TRIVIAL and FORMULA**.

The CHEMDNER Corpus consists of 10,000 abstracts published in 2013 in top journals from chemistry-related disciplines [18]. Each abstract selected was human annotated for all chemical mentions sufficiently specific to be able to be associated with chemical structure information. Nearly all mentions were assigned one of seven different subtypes (ABBREVIATION, FAMILY, FORMULA, IDENTIFIER, MULTIPLE, SYSTEMATIC, and TRIVIAL) as illustrated in Figure 1. The corpus is divided into Training (3,500 abstracts), Development (3,500 abstracts) and Test (3,000 abstracts) sets. The full CHEMDNER corpus and the complete annotation guidelines are available for download (after free registration) [20].

The CHEMDNER Corpus was prepared for the recent CHEMDNER task at BioCreative IV [18]. This task was separated into two sub-tasks, the CEM (Chemical Entity Mention) subtask, which evaluates performance at the mention level, and also the CDI (Chemical Document Indexing) subtask, which evaluates performance at the abstract level.

### System description

Many successful named entity recognition systems have improved performance by exploiting the complementary strengths of multiple models [5]. This approach is an instance of the machine learning method of ensemble learning, and requires sufficient differences between the systems combined [21]. Here we combine two linear chain conditional random fields (CRF) models employing different tokenizations and feature sets. These models were prepared by independently adapting existing named entity recognition systems, in an attempt to take advantage of the performance improvements of ensemble learning. Model 1 is an adaptation of the BANNER named entity recognizer to chemicals [22]. Model 2 was created using CRF++ [23] by repurposing part of the tmVar system for locating genetic variants [24]. Both models also employ multiple post processing steps. We then use several strategies to combine the output of the two models for improved performance. The differences between the models are summarized in Table 1 and described in detail in the following sections.

**Table 1:** Comparison of Model 1 and Model 2.

Aspect	Model 1	Model 2
System adapted	BANNER [22]	tmVar [24]

**Preprocessing**		

Unicode transliteration	No	Yes

Tokenization	whitespace punctuation digits lowercase to uppercase	whitespace punctuation digits lowercase to uppercase uppercase to lowercase

Sentence segmentation	Java BreakIterator	None

**Conditional random field configuration and settings**		

Implementation	MALLET [25]	CRF++ [23]

Order	1	2

Label model	IOB with one entity label	IOB with one entity label

Regularization	L_2_	L_2_

Gaussian prior variance (σ)	1.0	4.0

Feature frequency threshold	0	3

**Features**		

Individual tokens	Yes	Yes

Morphology	Lemmatization	Stemming

Part of speech	Yes	No

Word shapes	Yes	Yes

Characters	N-grams length 2 - 4	Prefixes and suffixes length 2 - 5

Character counts	None	Total characters, digits, uppercase, lowercase

ChemSpot [4]	Yes	No

Semantic affixes	None	Suffixes, alkane stems, trivial rings, simple multipliers, etc.

Chemical elements	Name and symbol	Name

Amino acids	Name, 3-char abbreviation, 1-char abbreviation	None

Chemical formulas	Within a single token	None

Amino acid sequences	Across tokens	None

Context window	2	3

**Post processing**		

Consistency	Yes	No

Abbreviation resolution	Yes	Yes

Parenthesis balancing	Yes	Yes

Chemical identifiers	Yes	Yes

This table compares the setup and configuration of Model 1 and Model 2.

### CRF model 1

**Figure 2:**
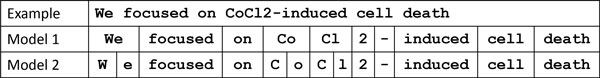
**Sample text illustrating tokenization differences between Model 1 and Model 2**.

Model 1 is an adaptation of BANNER [22], which is built on the MALLET toolkit [25], to chemical entities. Sentence segmentation is performed by the built-in Java class BreakIterator. The tokenization is finer than typically used for genes or diseases, breaking tokens not only at white space and punctuation but also between letters and digits and also between lower case letters followed by an uppercase letter. An example of the tokenization is provided in Figure 2. We used the IOB label set with only one entity label ("CHEMICAL"), a CRF order of 1, and L_2 _regularization using the default Gaussian prior variance, 1.0.

The feature set combines both features previously used in BANNER and additional features added to improve performance on chemicals. These additional features were developed through many rounds of iterative design involving training the model and analysing the results on our internal evaluation set. We describe the set of features as follows:

• Individual tokens and lemmas: We include a series of binary features indicating whether the token matches any token seen in the training data. We also include a series of binary features for the lemma for the token.

• Part of speech: We include a series of binary features for each part of speech.

• Word shapes: we process the token to inform the model about its pattern of letters and digits. All uppercase letters are replaced by "A," lowercase letters by "a," digits by "0" and all other characters by "x." The results are used as a binary feature.

• Capitalization patterns: We employ several binary features to indicate whether the token matches one of several capitalization patterns, such as all capital letters.

• Roman numerals and Greek letters: These are binary features which recognize if the token represents either a Roman numeral (e.g. "III") or the name of a Greek letter ("alpha").

• Character n-grams of length 2 through 4: Chemicals are rich in morphemes that are either semantically meaningful, rare in text outside of chemistry, or both. We therefore include character n-gram features of length 2 through 4, which is longer than typically used for genes and diseases.

• ChemSpot: The output of the ChemSpot system is used as a feature [4], providing functionality similar to a lexicon but with increased flexibility. We implement this as a binary feature, with any token overlapping a mention found by ChemSpot receiving the value 1, and all others 0.

• Chemical elements: We use a list of element symbols ("Tc") and element names ("Technetium"), as found in [26], to create two binary features. These features recognize whether a token matches the name of a chemical symbol or element, respectively.

• Amino acids: We also use a list of amino acid names and both 3-character and 1-character amino acid abbreviations, as found in [1], to create three binary features. These features respectively recognize whether a token matches the name of an amino acid, one of the 3-character abbreviations or one of the 1-character abbreviations.

• Chemical formulas: We determined that chemical formulas were represented in text in ways that were too variable to capture with straightforward pattern matching. Instead, we decided to allow the machine learning model handle the recognition of formulas statistically. To facilitate this, we used a pattern to identify tokens consisting of a sequence of element symbols, such as "FCH," the first token in the formula "FCH2CH2N3."

• Amino acid sequences: We used a pattern to recognize sequences of amino acids such as "Phe-Cys-Tyr," which crosses multiple tokens.

The total number of feature weights used in Model 1 is 794,979.

### CRF model 2

We developed a second CRF model using the CRF++ library [23] by repurposing part of the tmVar system for locating genetic variants [24]. Since the system which provided the initial implementation for Model 2 had a significantly different purpose and implementation than Model 1, the final models also contained significant differences.

Non-ASCII Unicode characters are transliterated to a similar ASCII equivalent, such as converting Greek alpha ("α") to the letter "a." No sentence segmentation is used. The tokenization is the same as tmVar; specifically, tokens are separated at whitespace and punctuation, digits, lowercase letters and uppercase letters are divided into separate tokens [24]. An example of the tokenization is provided in Figure 2. This model also uses the IOB label set, but uses an order 2 CRF model. It also uses L_2 _regularization, with a Gaussian prior variance set to 4.0. The feature cut-off threshold was set to 3, meaning that features that do not appear at least three times in the training data are not used.

This model was adapted iteratively through analysis of the evaluation set. Since the initial system is different than BANNER, the final resulting feature set is different than Model 1, as described below:

• General linguistic features: We included the original token and stems using the Porter stemmer [27].

• Prefixes and suffixes: We extracted the prefixes and suffixes (length: 1 to 5) as features.

• Character features: IUPAC mentions (e.g., "3-(4,5-dimethyl-thiazol-2-yl)-2-5-diphenyltetrazolium-bromide") include many digits and non-alphanumeric characters. For each token we therefore calculated several statistics, including the number of characters, number of digits and number of uppercase and lowercase letters, and use these as features.

• Roman numerals and Greek letters: These are binary features which recognize if the token represents either a Roman numeral (e.g. "III") or the name of a Greek letter ("alpha").

• Semantic features: We defined several binary features representing characteristics specific to chemicals, including suffixes (e.g. "-yl," "-oyl," "-one," "-ate," "acid," etc.), alkane stems (e.g. "meth," "eth," "prop" and "tetracos"), trivial rings (e.g. "benzene," "pyridine" and "toluene") and simple multipliers ("di," "tri" and "tetra"), as derived from [28].

• Chemical elements: We included a binary feature to recognize the names of the chemical elements (e.g. "hydrogen") [26].

• Case pattern features: We applied the case pattern features from tmVar [24]. Each token is represented in a simplified form. Upper case alphabetic characters are replaced by "A" and lower case characters are replaced by "a." Likewise, digits (0-9) are replaced by "0." Moreover, we also merged consecutive letters and numbers and generated additional single letter "a" and number "0" as features.

• Contextual features: We included the general linguistic and semantic features of three tokens from each side as context. This is larger than for Model 1, which uses only two tokens on each side.

Our analysis of the results of ChemSpot on our internal evaluation set demonstrated good performance for long chemical mentions but relatively low recall for the short mentions that constitute the majority of the annotations in the CHEMDNER corpus. However our analysis of Model 2 already demonstrated good performance on long chemical mentions, and strong features such as the output of a system like ChemSpot tend to strongly bias conditional random field models. Thus, we did not use the output of ChemSpot as a feature for Model 2, even though it helped Model 1, and this difference helps to preserve the independence of the two models. The total number of feature weights used in Model 2 is 96,435,808, two orders of magnitude more than in Model 1.

### Post-processing methods

We employed several post-processing steps, which varied slightly between Models 1 and 2. These steps include enforcing tagging consistency, abbreviation resolution, boundary revision to balance parenthesis, and recognizing identifiers.

We improved consistency - and significantly improved recall - by tagging all instances of a specific character sequence as a chemical mention if that sequence was tagged by the CRF model at least twice within an abstract. For example, if the CRF model found two instances of "steroidal saponins," a FAMILY mention, within an abstract but missed a third instance, the missed instance would be added. We further modified this module after the CHEMDNER task to not add mentions that overlap with mentions already tagged, for example to not add a tag for "GO" if "GO-PDEA" is already tagged. This module was only used by Model 1, since the output of Model 2 was found to be already be sufficiently consistent that this rule did not improve performance.

We used the Ab3P tool to find local abbreviation definitions, such as "allopregnanolone (AP)" [29]. In both models, if the long form was tagged by the CRF model, then all instances of the abbreviation would be tagged in the result. Model 2 employed two additional rules that were not found to help Model 1. First, if both the abbreviation and long form mention were tagged by the CRF model, then all mentions of the long form would be tagged. Second, if the long form mention was not tagged, then mentions matching the abbreviation were removed.

While there are several mentions in the training data with unbalanced parenthesis, square brackets and curly brackets (braces), we determined that virtually all of the unbalanced mentions returned by either of our models were errors. We therefore attempt to balance each mention with respect to parenthesis, square brackets and curly brackets (braces) by adding or removing one character to the right or left of the mention. For example, if the model tags "Cu(2+" and the next character in the text is a right parenthesis (")"), then the mention is extended to include it. If no variant of adding or removing one character to the right or left results in balanced parenthesis, we simply drop the mention. This module is used by both Models 1 and 2.

Chemical identifiers do not have a specific format, making them very difficult to identify with machine learning. We therefore created a lexicon of chemical identifiers from the CTD database http://ctdbase.org/ by extracting the chemical names consisting of 2 to 5 letters, followed by at least two digits. We apply these as patterns, allowing the characters between the letter and digit blocks to vary. For example, the lexicon name "NSC-114792" becomes the regular expression "NSC[\-\_ ]{0,2}114792". This module is also used by both Model 1 and 2.

### Converting CEM results to CDI results

The CDI task requires informative confidence rankings for each mention. Model 1 used an approximation of the marginal probability for each mention, that is, the probability that the mention is correct, to calculate the probability that the mention appears at least once in the abstract.

Unfortunately, the MALLET implementation of marginal probability only provides the marginal probability of each label. We therefore approximate the marginal probability of each mention using *n*-best inference, which determines the *n *label sequences with the highest joint probability [30]. We find the set of mentions present in the *n *= 20 label sequences with the highest joint probability, and then consider the marginal probability of each mention to be the sum of the joint probabilities of the label sequences where the mention appears.

To calculate the probability that the mention appears at least once in the abstract, we employ an assumption that each mention is independent of the others, and apply the *noisy or *function, which combines the probability that the mention appears with its frequency within the abstract in a natural way [31]. Specifically, given that a text *t *is found as a mention *n *times in abstract *a*, with mention-level probabilities $p_{1}^{m}\left(t\right)$ through $p_{n}^{m}\left(t\right)$, the abstract-level probability for t, *p^a^*(*t*), is calculated as:

$$p^{a}\left(t\right)=1-\overset{n}{\underset{i=1}{\prod }}\left(1-p_{i}^{m}\left(t\right)\right)$$

CRF++ does not provide implementations of either marginal probability or *n*-best decoding, so Model 2 instead uses a small fixed probability.

### Model combinations

We take advantage of the differences between Model 1 and Model 2 to combine their results in three different ways. The "naïve combination" merely pools the results from Model 1 and Model 2. We noted during analysis of the evaluation set that Model 2 had higher performance on short mentions, and consequently provide a "heuristic combination" which uses the results from Model 1 but replaces mentions of length 5 to 15 with the corresponding results from Model 2. Our "high recall combination" combines the mentions returned by n-best decoding in Model 1 with at least a marginal probability of 0.1 - which provides high recall while maintaining reasonable precision - with the results from Model 2.

### Normalization

While recognizing chemical mentions is valuable, many tasks ultimately require the mention to be identified or normalized. We have thus paired our named entity recognition system with a straightforward lexical approach for normalization. Our lexicon of chemical entities and their names was collected from MeSH [32] and ChEBI [33]. The system converts both mentions from the literature and entity names in the lexicon to lowercase and removes all whitespace and punctuation. For example, "flavone-C-glycoside" becomes "flavonecglycoside." The system then assigns a MeSH identifier to those mentions which can be found in the lexicon, or a ChEBI identifier if a matching MeSH identifier cannot be found. Mentions that correspond to a short form recognized by Ab3P are assigned the same identifier as the long form found by Ab3P [29]. Mentions which do not map to a specific identifier are ignored and mentions which can be assigned to both a MeSH and ChEBI identifier are only assigned the MeSH identifier.

## Results

The evaluation measures consisted of precision (the proportion of mentions returned that are correct), recall (the proportion of correct mentions that are returned) and f-measure (the harmonic mean of precision and recall). In the CEM task, a true positive (*tp*) consists of the system returning a mention whose boundaries exactly match the boundaries of a mention annotated in the test data. False positives (*fp*) are defined as the system returning a mention whose boundaries do not match any mention annotated in the test data, and false negatives (*fn*) are defined as a mention annotated in the test data whose boundaries do not match any mention returned by the system. The performance measurements for the CDI task are defined similarly, except that instead of requiring mention boundaries to match, the match must be the mention texts. Given these definitions, precision (*p)*, recall (*r*) and f-measure (*f*) are defined as:

$$p=\frac{tp}{tp+fp}\;r=\frac{tp}{tp+fn}\;f=2\frac{p\cdot r}{p+r}$$

We report two sets of performance values: one on our internal evaluation set and one on the official test set. The internal evaluation set was created by pooling the official training and development data, then randomly splitting this pool into 6000 abstracts for training (internal training set) and 1000 abstracts for evaluation during development (internal evaluation set). The performance values for the internal evaluation set were created by training each model on the internal training set and evaluating on the internal evaluation set. The performance values for the official test set were created by training each model on the union of the official training and development sets, then evaluating on the official test set. Overall results for the CEM task are reported in Table 2 and results per chemical entity subtype are reported in Table 3. Overall results for the CDI task are reported in Table 4.

**Table 2:** Results for CEM task.

Setup	Internal Evaluation Set			Official Test Set		
	
	P	R	F	P	R	F
Model 1	0.8773	0.8758	0.8766	0.8595	0.8721	0.8657

Model 2	**0.8781**	0.8634	0.8707	**0.8909**	0.8575	**0.8739**

Naïve combination	0.8323	**0.9290**	0.8780	0.8192	0.9209	0.8671

Heuristic combination	0.8659	0.9002	**0.8827**	0.8516	0.8906	0.8706

High recall combination	0.7651	**0.9290**	0.8391	0.7672	**0.9212**	0.8372

Results for the CEM task for each experimental setup on our internal evaluation set and the official test set, as measured by micro-averaged precision (P), recall (R) and f-measure (F). The highest value is shown in bold.

**Table 3:** Results for CEM task per chemical entity subtype.

Setup	ABBR	FAMI	FORM	IDEN	MULT	SYST	TRIV	NONE
Model 1	0.8768	0.8216	0.8199	0.8323	0.3969	0.9136	0.9013	0.7073

Model 2	0.8285	0.7871	0.8393	0.8615	0.4824	0.9196	0.8783	0.6829

Naïve combination	0.9132	0.8799	0.8936	0.9005	0.5326	0.9588	0.9403	0.7804

Heuristic combination	0.8837	0.8440	0.8318	0.8986	0.4020	0.9276	0.9273	0.7804

High recall combination	0.9137	0.9006	0.8809	0.8752	0.6030	0.9525	0.9414	0.7560

Results for the CEM task for each experimental setup on each chemical entity subtype, as measured by micro-averaged recall.

**Table 4:** Results for CDI task.

	Internal Evaluation Set			Official Test Set		
	
Setup	P	R	F	P	R	F
Model 1	**0.8732**	0.8664	0.8698	**0.8804**	0.8606	0.8704

Model 2	0.8572	0.8806	0.8687	0.8781	0.8724	**0.8752**

Naïve combination	0.8138	**0.9270**	0.8667	0.8268	0.9189	0.8704

Heuristic combination	0.8565	0.8934	**0.8745**	0.8663	0.8825	0.8743

High recall combination	0.7422	**0.9270**	0.8244	0.7629	**0.9224**	0.8351

## Discussion

There is a notable difference in the results between the evaluation set and the test set. The results on the evaluation set followed the expected trends. Model 1 and Model 2 have similar performance. The naïve model combination of the Model 1 and Model 2 results improves recall at the expense of precision, the heuristic combination in provides the highest f-measure, and our high recall combination provides the highest recall. The primary difference in the test set is a reduction in precision for Model 1, resulting in a reduction in f-measure by over 1%. While a performance reduction of this magnitude is not unusual, this contrasts with Model 2, where an increase in precision causes the f-measure to increase. These trends carry over into the combination runs. The difference between the CDI results are smaller than the difference between the CEM results, presumably due to the use of the marginal probability in Model 1. The f-measure achieved by Model 2 on the CEM task was the highest achieved by any submission to the CHEMDNER task.

### Error analysis

In other entity types such as genes, proteins and diseases, determining the entity type of tokens not observed in the training set is frequently difficult and must often rely on context. Many tokens in chemical mentions have highly distinctive features, however, which frequently allows the model to infer that the token is part of a chemical mention even if the token has not been seen previously. The greater problem for chemicals seems to be determining the mention boundaries, where errors cause a significant performance reduction since each boundary error results in both a false positive and a false negative under the common practice of assigning each token to at most one mention. We found, for example, that allowing boundaries to overlap instead of match exactly resulted in an f-measure for Model 1 of over 0.92.

One issue which causes boundary errors in many entity types is modifiers, since whether the modifier is considered part of the mention or not depends on the both the modifier and the core term. Our error analysis found many cases of boundary errors due to modifiers, such as returning "aromatic hydrocarbons" instead of "polycyclic aromatic hydrocarbons" or returning "Gynostemma pentaphyllum saponins" instead of "saponins." We anticipate that improving the modelling of tokens not seen during training could address these cases.

Another issue common in other entity types is mentions containing coordination ellipsis [34], such as "oleic, linoleic, and palmitic acids." We observed both models having difficulties finding complete coordinations, instead, we often observed the models locating the non-elliptical part of the mention (such as "3-O-alkyl clarithromycin" from the mention "3-hydroxyl, 3-O-acyl and 3-O-alkyl clarithromycin"). It seems likely that existing methods to handle coordination based on supervised machine learning, such as [35], would be effective in many of these cases. However, we believe that fully effective coordination resolution may be a considerable challenge due to the significant complexity of many coordinated chemical mentions (e.g. "ribo and 2'-b-C-methyl ribo Janus type nucleosides"). These mentions may benefit from a method that combines coordination resolution with a lexical approach, such as [34].

Another phenomenon more specific to chemicals relates to the use of systematic and formula names. While some chemical names - typically TRIVIAL names - are relatively short and possess comparatively unambiguous boundaries, chemical formulas and systematic names are descriptive terminologies whose productivity mirrors the infinite array of possible chemicals. Since lists of chemicals and chemical compounds are annotated as separate mentions, the classifier must decide after every token whether to extend the current mention or whether the next token begins a new mention. While one would expect some subtypes to be more fixed (e.g. TRIVIAL and FAMILY) and other to trend more toward extensibility (e.g. SYSTEMATIC and FORMULA), we instead observed that the annotated chemical subtypes did not provide sufficient information to allow the models to reliably differentiate between them. For example, some mentions annotated as subtype FAMILY trend towards extensibility (e.g. "2-acetamido-3-mercapto-3-methyl-N-aryl-butanamides") while some SYSTEMATIC mentions would be considered as relatively fixed phrases (e.g. "phthalate"). The primary need for handling this problem is to provide some way to model mention completeness. This could be handled in many ways. A lexical approach would attempt to ensure completeness of chemical entity mentions by referring to a lexicon of chemical entities. A rule-based method would provide the same information, but may allow IUPAC and other systematic nomenclatures to be recognized directly. These methods both model mention completeness indirectly by determining the identity of the chemical entity mentioned. An unsupervised method to model completeness directly might attempt to locate the same mention in a large amount of unlabelled text, where the boundaries may be less ambiguous [36,37].

Non-boundary errors were of several types. One significant source of error is short proteins. Because proteins are large molecules with specific functions, they are essentially highly specialized chemicals. However the CHEMDNER task defined chemicals from the structural composition perspective, specifically defining peptides 15 amino acids or longer as biochemical entities, which are not annotated. Thus, relatively short proteins such as "kaliotoxin" and "thioredocin-1" are often mentioned in the abstracts with a discussion of their uses and effects, similar to chemicals, but are considered false positives when found because their length in amino acids exceeds the threshold set for annotation. Since determining if these mentions are chemicals requires integrating knowledge that is often not present in the abstract, resolving these cases requires identifying the mention, suggesting that named entity recognition and normalization are not always independent steps.

Abbreviations are highly ambiguous, and while our use of abbreviation post-processing significantly helped with their recognition, we found that Ab3P was not as successful at locating the full mentions as in our recent work on diseases [9]. The primary reason appears to be non-alphabetic characters at the beginning of the long form of the name, such as "1,2,3,4,6 penta-O-galloyl-b-d-glucose (PGG)." We anticipate that updating the abbreviation model to anticipate non-alphabetic content at the beginning of the long form which is not reflected in the short form would largely resolve this issue.

### Differences between the models

We performed a series of experiments to explore which differences between Model 1 and Model 2 caused the performance differences between the two models. In these experiments we chose the aspects of Model 2 we considered most likely to improve the performance of Model 1. These differences were then implemented in Model 1, applied one at a time, and performance recalculated. To clarify exposition, we describe these differences in two sets.

The first set of differences we considered were the finer tokenization, the model order (order 2 instead of 1), the size of the feature context window (3 instead of 2), and the Unicode transliteration preprocessing. All experiments from this set resulted in a reduction to both precision and recall on both the Evaluation and Test sets (data not shown).

The second set of differences we considered were the Gaussian prior variance and the feature frequency threshold. The Gaussian prior variance (σ) controls the strength of the regularization, so that reducing σ lowers the ability of the model to fit the data, increasing generalization and decreasing the chance of overfitting. During the task, Model 1 used σ = 1.0, the default, while Model 2 used σ = 4.0. The feature frequency threshold (c) specifies that any feature appearing in a positive context fewer times than the threshold would not be included in the model. Model 1 did not use a feature frequency threshold during the task (effectively 0), while the threshold was set to 3 for Model 2. Increasing the frequency threshold should result in increased model stability, though this may come at the expense of ignoring some useful rare signals.

Since a model with more stable features should require less regularization, we expected that it would be useful to optimize σ and c together. We therefore ran a series of 16 experiments, jointly varying the values of σ and c in Model 1 between σ = {0.5, 1.0, 2.0, 4.0} and c = {0, 1, 3, 5}. Contrary to our expectation, we found no clear relationship between either σ or c and the resulting performance (data not shown). We did determine, however, that performance on the Evaluation set was generally predictive of performance on the Test set. The highest precision on the Evaluation set was provided by σ = 1.0 and c = 0 - the default configuration - and the highest recall and f-measure were provided by σ = 2.0 and c = 1. While these configurations also performed very well on the Test set, the configuration with the highest f-measure on the Test set was σ = 0.5 and c = 5, primarily due to unexpectedly high precision. The performance of these configurations is described in Table 5.

**Table 5:** Model 1 CEM results for optimizing Gaussian prior variance and feature frequency threshold.

	Internal Evaluation Set			Official Test Set		
	
Setup	P	R	F	P	R	F
σ = 1.0, c = 0	**0.8773**	0.8758	0.8766	0.8595	**0.8721**	0.8657

σ = 2.0, c = 1	0.8758	**0.8778**	**0.8768**	0.8973	0.8474	0.8716

σ = 0.5, c = 5	0.8766	0.8731	0.8749	**0.9009**	0.8462	**0.8727**

Model 1 results for the CEM task for the Gaussian prior variance (σ) value and feature frequency threshold (c) value, which resulted in the highest micro-averaged precision (P), recall (R) and f-measure (F) on our internal evaluation set and the official test set. The highest value is shown in bold.

Results for the CDI task for each experimental setup on our internal evaluation set and the official test set, as measured by micro-averaged precision (P), recall (R), and f-measure (F). The highest value is shown in bold.

These results suggest that jointly optimizing σ and c is useful and that the best advice is to use the configuration that performs best on the Evaluation set. It should also be noted, however, that the difference between the highest and lowest result for precision, recall and f-measure (after removing the configuration σ = 0.5, c = 0, which performed poorly) are all relatively small, approximately 0.01 (data not shown).

While these experiments have not exhaustively tested every difference between the two models, most of the differences between Model 1 and Model 2 caused a reduction in performance when ported to Model 1. This result suggests that the models are in fact independent. The differences which improved performance - optimizing the feature frequency threshold (c) and the regularization parameter (the Gaussian prior variance, σ) allowed Model 1 to approach the performance of Model 2, however.

## Conclusions

We used a model combination approach where the two models have many differences. The models used different tokenizations, feature sets, CRF implementations, CRF parameters, and some variations in post processing. This is in contrast to most previous NER work on model combination, where many models are used but they typically differ in only a single aspect, such as the model order [30]. Many of the remaining differences reduce performance when the models are modified to be more alike.

Model 1 experienced a slight drop in performance between the Evaluation and Test sets, while the performance of Model 2 increased slightly. Model 2 achieved the highest f-measure reported on the CEM task. Our model combination runs succeeded in providing higher recall, and we found our approach of preparing and combining multiple CRF models and post-processing to be effective overall. Unfortunately, combining the results of multiple models incurs some inconvenience for practical use, and the large size of the file containing the trained parameters for Model 2 (over 1 Gb) may limit widespread use. Regardless, both Model 1 and Model 2 are available at our supplementary website (see abstract for the URL). Moreover, the results of a full run of Model 2 over PubMed is available in PubTator [6,38], including normalization using the lexical approach described in the Methods section.

We note that tmChem is now able to report performance for mention-level detection of chemicals competitive with (or even greater than) the performance typically reported for genes, proteins and diseases. In addition, our error analysis uncovered several problems where further development would likely improve performance. Interestingly, most of these would clearly benefit from providing the named entity recognition step with an informative signal regarding the identity of the chemical entity being mentioned. Like gene and other concept recognition tasks [9,39], it is important to investigate how to normalize detected chemical mentions to standard terminologies or ontologies in future studies.

## Authors' contributions

Conceived and designed the experiments: RL, CHW and ZL. Performed the experiments: RL and CHW. Drafted the manuscript: RL, CHW and ZL. All authors read and approved the manuscript.

## Competing interests

The authors declare that they have no competing interests.

## Declarations

This research and publication was supported by the Intramural Research Program of the NIH, National Library of Medicine. The authors declare that they have no competing interests.

This article has been published as part of *Journal of Cheminformatics *Volume 7 Supplement 1, 2015: Text mining for chemistry and the CHEMDNER track. The full contents of the supplement are available online at http://www.jcheminf.com/supplements/7/S1.
